# Supplementary material for: Laboratory disruption of scaled astrophysical outflows by a misaligned magnetic field
Source: Nat Commun. 2021 Feb 3;12:762. doi: 10.1038/s41467-021-20917-x (PMC7858631; doi:10.1038/s41467-021-20917-x)
Supplement: Supplementary file 1 — Supplementary Information [file 41467_2021_20917_MOESM1_ESM.pdf]

Supplementary Information: Laboratory disruption of scaled astrophysical outflows by a misaligned magnetic field

G. Revet,[1,2,3] B. Khiar,[4,5] E. Filippov,[1,6] C. Argiroffi,[7,8] J. Béard,[9] R. Bonito,[8] M. Cerchez,[10] S. N. Chen,[1,11] T. Gangolf,[10,2] D. P. Higginson,[2,12] A. Mignone,[13] B. Olmi,[14] M. Ouillé,[2] S. N. Ryazantsev,[15,6] I. Yu. Skobelev,[6,15] M. I. Safronova,[1] M. Starodubtsev,[1] T. Vinci,[2] O. Willi,[10] S. Pikuz,[6,15] S. Orlando,[8] A. Ciardi,[4] and J. Fuchs[1,2]

[1]*Institute of Applied Physics, 46 Ulyanov Street, 603950 Nizhny Novgorod, Russia*
[2]*LULI, CNRS, CEA, Sorbonne Université, École Polytechnique, Institut Polytechnique de Paris, F-91128 Palaiseau, France*
[3]*Centre Laser Intenses et Applications, Université de Bordeaux-CNRS-CEA, UMR 5107, 33405 Talence, France*
[4]*Sorbonne Université, Observatoire de Paris, PSL Research University, LERMA, CNRS UMR 8112, F-75005, Paris, France*
[5]*Flash Center for Computational Science, University of Chicago, 5640 S. Ellis Avenue, Chicago, IL 60637, USA*
[6]*Joint Institute for High Temperatures, RAS, 125412, Moscow, Russia*
[7]*Dipartimento di Fisica e Chimica, Universitá di Palermo, Palermo, Italy*
[8]*INAF–Osservatorio Astronomico di Palermo, Palermo, Italy*
[9]*LNCMI, UPR 3228, CNRS-UGA-UPS-INSA, 31400 Toulouse, France*
[10]*Institut für Laser und Plasmaphysik, Heinrich Heine Universität Düsseldorf, D-40225 Düsseldorf, Germany*
[11]*ELI-NP, "Horia Hulubei" National Institute for Physics and Nuclear Engineering, 30 Reactorului Street, RO-077125, Bucharest-Magurele, Romania*
[12]*Lawrence Livermore National Laboratory, Livermore, California 94550, USA*
[13]*Dip. di Fisica, Universiá di Torino via Pietro Giuria 1 (10125) Torino, Italy*
[14]*INAF-Osservatorio Astrofisico di Arcetri, Firenze, Italy*
[15]*National Research Nuclear University 'MEPhI', 115409 Moscow, Russia*

SUPPLEMENTARY NOTE 1: ESTIMATION OF THE YSO'S AMBIENT MAGNETIC FIELD

In order to infer the ambient magnetic field in the surrounding of the YSO's outflow, we use the observed value of the collimation radius: i.e. the radius for which the YSO jet is not showing a strong divergent expansion anymore, but displays instead a thermal expansion compatible with the expected radial expansion of a supersonic collimated jet [1, 2]. In the case of the DG Tau A object and its associated HH 158 jet for instance, this radius is $R_{\rm c} \sim 15$ AU [3], at a distance of 50 AU from the outflow source.

By equalizing the ram pressure of a YSO expanding ejecta ($P_{\rm out} = \dot{M}_{\rm out} \times v_{\rm out}/S$) with the magnetic pressure it is subjected to ($P_{\rm mag} = B^2/8\pi$), it is possible to show that such collimation radius $R_{\rm c}$ of the jet (i.e. the cavity border location, where $P_{\rm out} = P_{\rm mag}$), is $R_{\rm c} = (2 \times \dot{M}_{\rm out} \times v_{\rm out})^{1/2} B^{-1}$ [4] ; where B is the magnetic field, $\dot{M}_{\rm out} = \rho \times v_{\rm out} \times S$ the ejected mass rate passing through the sphere surface $S = 4\pi R^2$ and $v_{\rm out}$ the speed of the ejecta.

This leads in practice, within the distance-from-the-source region $10 < z < 50$ AU, for a flow velocity of 400 km s^{-1} [5] and an ejected mass rate of 10^8 M$_\odot$ yr^{-1} [3, 5] to a magnetic field of $B = 24$ mG necessary for shaping the outflow within the observed collimation radius of $R_{\rm c} \sim 15$ AU [3].

SUPPLEMENTARY NOTE 2: X-RAY SPECTROSCOPY MEASUREMENT

Complementarily to the optically-measured electron density maps shown in Fig.1 to 3 of the main text, the electron temperature and dynamics of jet expansion were also studied by means of X-ray spectroscopy, looking at the He-like and H-like Fluorine ions.

The 2D X-ray maps presented in Fig.2(d, e, f) of the main text support the optical interferometry data, in particular as they show the same phenomenon: the plasma initially expands normal to the source target, but later on bends, finally following the magnetic field lines direction. They also show that the dense part of the plasma flow, dense enough to yield detectable X-ray emission, is much shortened when α increases.

In Fig.2(d, e, f), only the images retrieved from the F He 4p-1s line emission are presented, however we can state that other observed spectral lines reveal a quite similar behavior. We note that the overall plasma evolution features and distances of sustained propagation, as retrieved from the interferometry diagnostic and the X-ray images, when the jet is observed to be deflected by the magnetic field, are similar. Thus, both diagnostics concur to demonstrate that the initial plasma expansion occurs normal to the target surface and is identical to the case with no B field. Also, both diagnostics observe that the final jet propagation direction is imposed by the B field orientation and not by the initial outflow expansion axis.

The electron temperature along the plasma jet axis

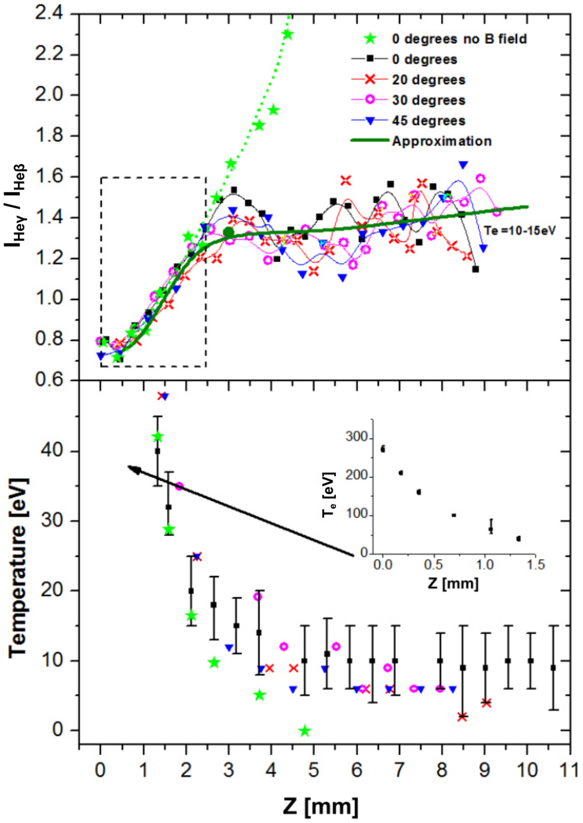

Supplementary Figure 1. (top) Fluorine Heγ to Heβ lines intensity ratio measured along the jet axis for different orientations of the magnetic field, i.e. 0, 20, 30 and 45 degrees, with respect to the plane of the target and also in the case when no magnetic field is applied. The dashed rectangle delimits the range where the plasma ram pressure dominates the external B field pressure, and where the line ratios are observed to be equal for all cases, as expected. (bottom) Corresponding time-integrated electron temperature of the jet plasma and as revealed by means of our recombining plasma model method. The inset corresponds to the region near the target surface where the temperature is above 50 eV. The error bars shown for the 0 degrees case apply to all cases; they are shown only for the 0 degrees case for clarity. Those error bars are due to intrinsic instrumental x-ray spectrometer resolution limits as well as the data analysis method used - for precise details about this analysis method and the error bars definition, the reader should refer to the following paper [6].

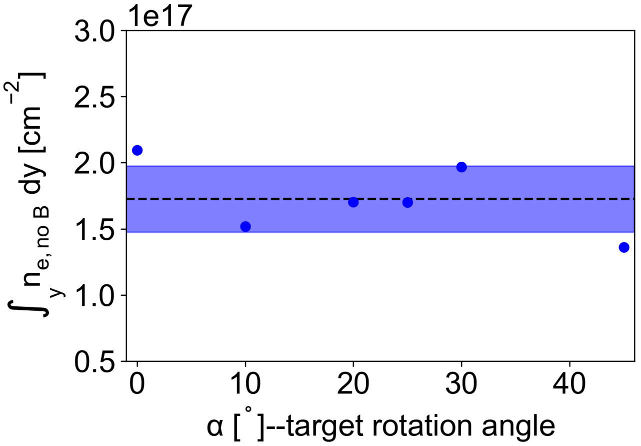

Supplementary Figure 2. Fluctuation of the integrated plasma density at the location $z_{c.m.} = 5$ mm in the absence of external magnetic field and for different target angles, at 16 ns after the laser interaction. The blue rectangle displays the standard deviation, σ, of the set of points measured for different angles with respect to the constant defined as the average of the points (materialized by the dashed horizontal line).

was determined by analyzing the relative intensities of Heγ to Heβ spectral lines. The method [7] is based on the quasi-steady model of expanding plasmas which also takes into account a recombining plasma model with a "frozen" ion charge. The results are shown in Supplementary Figure 1. While the electron temperature T_e on the laser irradiated target surface is about 300 eV, it drops down to 20 eV at a 2.5 mm distance from the target surface. Over this range, the lines intensity ratios and, correspondingly, the temperatures are almost the same regardless of the direction of the applied external magnetic field, or even of the existence of the latest. In the initial plasma expansion zone, this can be understood as the ram pressure strongly prevails over the magnetic field pressure. Beyond this initial expansion, the presence of the magnetic field allows in a case of perfectly aligned field and plasma expansion to keep the temperature of the formed jet almost constant in the range of 10 ± 5 eV at least for 8 mm long, consistently with our previous observations [7–9]. This is significantly different from the case without magnetic field, in which the temperature of the jet rapidly drops below 5 eV, as the density decreases to values smaller than 10^{18} cm^{-3}, i.e. beyond a distance of 4 mm from the target (see Supplementary Figure 1). In the presence of the magnetic field, our measurements show that similar temperatures are recorded when we vary the direction of the applied magnetic field. However, we have to note that our method is not sensitive enough in the range of 0 to 10 eV temperatures, due to very low signal to noise ratio in the spectral line intensity data. Hence, although we can see that the plasma certainly stays hotter when the magnetic field is applied than without it, we can not determine with high precision significant temperature differences in the cases where the magnetic field orientation is modified.

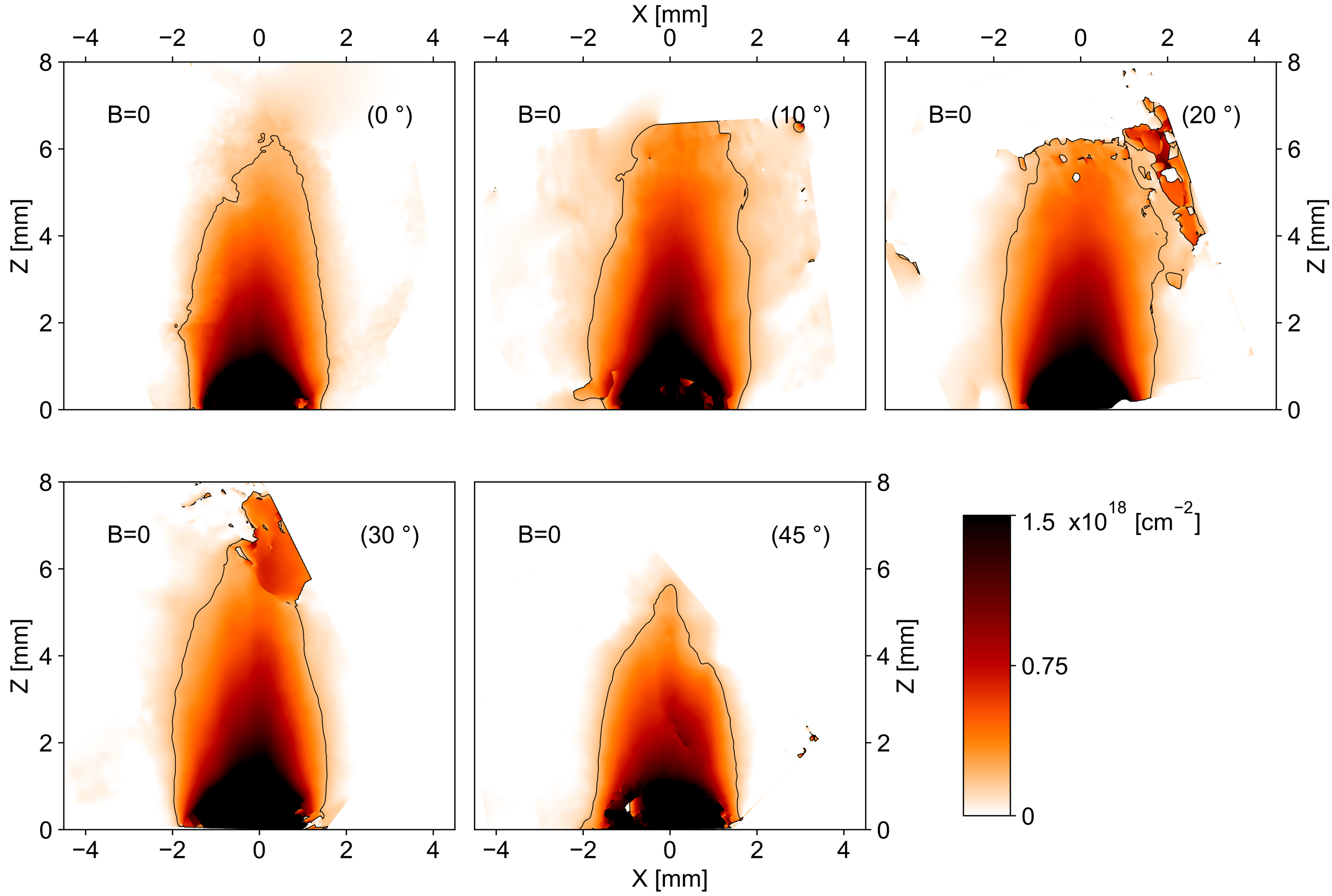

Supplementary Figure 3. Experimental integrated density of plasma expansion recorded for different target angles, at 16 ns after the laser interaction and in the absence of external magnetic field. The black lines follow the contour of the integrated electron density of 2×10^{17} cm^{-2}.

SUPPLEMENTARY NOTE 3: STABILITY OF THE LASER-SOLID INTERACTION AND PLASMA EXPANSION REGARDING THE TARGET ORIENTATION WITH RESPECT TO THE HIGH-POWER LASER

In order to examine the effect of the target angle on the plasma expansion, we look at the variation of the plasma density at the location $z_{c.m.} = 5$ mm for cases without any magnetic field applied and for different target angles (and so different laser shots). This is summarized in Supplementary Figure 2. As can be observed, fluctuations around the mean value indicated by the dashed line are moderate and no increasing or decreasing trend is observed in the plasma density when varying the target angle. Thus, a fluctuation around a constant value seems to adequately describe the density at a given distance in the unmagnetized case. This fluctuation is defined by one standard deviation, σ, of the distribution of points (blue circles) which are represented in Supplementary Figure 2. The one standard deviation is materialized by the blue rectangle in the same figure. This fluctuation is attributed to the laser energy shot-to-shot fluctuations.

In Fig.4(f) of the main text, the full triangles use a single value for the cases without magnetic field, whatever the angles, which is taken as the mean value (marked by the horizontal dashed line in Supplementary Figure 2); the empty dots use instead, for each angle, the associated value for the without-magnetic-field-shot, i.e. the individual dots in Supplementary Figure 2.

We note here that the standard deviation, σ, over the constant value found here is taken as the typical uncertainty on the plasma density measurements. Then, the error bars of Fig.4(f) of the main text are calculated with this error for both, the density with and without magnetic field, at $z_{c.m.} = 5$ mm. Explicitly, for the data shown in Fig.4(f) of the main text (i.e. $\int_y n_{\mathrm{e,B}}\, dy \;/\; \int_y n_{\mathrm{e,no\ B}}\, dy$, which we can define as a ratio a/b), the error bars are calculated as follows: $\sigma/b \,+\, \sigma \times a/b^2$.

Also, the complete 2D integrated electron density maps at 16 ns, for different angles, are displayed in Supplementary Figure 3.

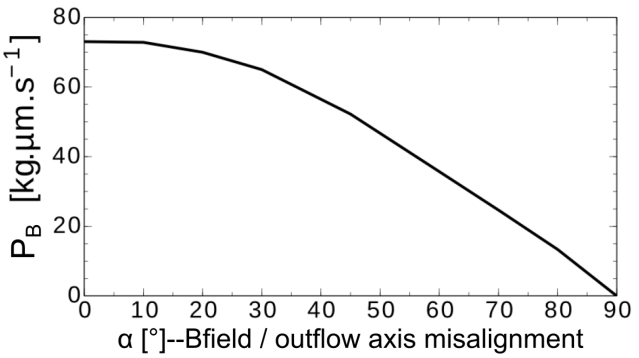

Supplementary Figure 4. Spatially and temporally averaged momentum of the plasma along the direction of the magnetic field as a function of the misalignment angle α, retrieved from the simulations shown in Fig.5 of the main text. The plasma momentum is averaged spatially over the full simulation domain ($6 \times 6 \times 13$ mm) and temporally over the full simulation duration (100 ns).

SUPPLEMENTARY NOTE 4: ADDITIONAL DISCUSSION FROM THE GORGON 3D-MHD SIMULATIONS

As illustrated by the Fig.6 of the main text, in the case of a large angle between the plasma flow and the magnetic field, the two components (cavity wall oblique shocks + cavity tip diamond shock) of the magnetic nozzle are unable to efficiently force the flow to align with the initial magnetic field direction over large scales. The plasma flow, at the tip of the cavity, is spread instead along the $x-$direction instead. This generates a plasma front, extended in the $x-$direction, which then actively reduces the bending of the magnetic field lines. Such a bending reduction, finally reduces drastically the ability of the magnetic forces to act against the plasma propagation. The plasma sheet, still quite hot and conductive, then pushes in the $z-$direction the magnetic field lines, frozen-in the plasma, in a much more favourable planar way. This has to be opposed to the initial plasma redirection at the cavity borders: the advection of the magnetic field is limited here, as a strong bending of the lines is observed, and the plasma is slowed down much more easily. Finally, the plasma sheet, while growing in size in the $x-$direction (reducing further the magnetic field lines bending), progresses in the $z-$direction ; as also attested by the declining trend of the plasma momentum aligned with the magnetic field lines.

Another observable, bringing to light the lack of collimation of the outflow with increasing the misalignment, is the momentum parallel to the local magnetic field, $P_{||\mathrm{B}}$, resulting from the whole process. Supplementary Figure 4 displays the evolution of such momentum with the misalignment. One can observe that $P_{||\mathrm{B}}$ stays similar between the perfectly aligned ($0°$) case (where the plasma flow is redirected along the magnetic field lines at best) and the $10°$ case, pointing out the efficient redirection of the plasma flow for such small misalignment. After this point however, $P_{||\mathrm{B}}$ starts to decrease and progressively reaches zero at an extreme misalignment of $\alpha = 90°$: the whole plasma then propagates perpendicularly to the magnetic field lines, advecting them in a perfectly planar way.

[1] Dougados, C., Cabrit, S., Lavalley, C. & Ménard, F. T Tauri stars microjets resolved by adaptive optics. *Astronomy & Astrophysics* **357**, L61–L64 (2000).

[2] Hartigan, P., Edwards, S. & Pierson, R. Going Slitless: Images of Forbidden-Line Emission Regions of Classical T Tauri Stars Observed with the Hubble Space Telescope. *The Astrophysical Journal* **609**, 261–276 (2004).

[3] Ainsworth, R. E., Ray, T. P., Scaife, A. M., Greaves, J. S. & Beswick, R. J. Subarcsecond high-sensitivity measurements of the DG tau jet with e-MERLIN. *Monthly Notices of the Royal Astronomical Society: Letters* **436**, 64–68 (2013).

[4] Matt, S., Winglee, R. & Böhm, K.-H. Collimation of a central wind by a disc-associated magnetic field. *Monthly Notices of the Royal Astronomical Society* **345**, 660–670 (2003).

[5] Maurri, L. *et al.* Physical properties of the jet from DG Tauri on sub-arcsecond scales with HST/STIS. *Astronomy & Astrophysics* **565**, A110 (2014).

[6] Filippov, E. *et al.* X-ray spectroscopy evidence for plasma shell formation in experiments modeling accretion columns in young stars. *Matter and Radiation at Extreme* **064402** (2019).

[7] Ryazantsev, S. N. *et al.* X-ray spectroscopy diagnostics of a recombining plasma in laboratory astrophysics studies. *JETP Letters* **102**, 707 (2016).

[8] Albertazzi, B. *et al.* Laboratory formation of a scaled protostellar jet by coaligned poloidal magnetic field. *Science* **346**, 325–8 (2014).

[9] Higginson, D. P. *et al.* Detailed characterization of laser-produced astrophysically-relevant jets formed via a poloidal magnetic nozzle. *High Energy Density Physics* **23**, 48–59 (2017).
